# A melanoma helper peptide vaccine increases Th1 cytokine production by leukocytes in peripheral blood and immunized lymph nodes

**DOI:** 10.1186/2051-1426-2-23

**Published:** 2014-07-15

**Authors:** Patrick M Dillon, Walter C Olson, Andrea Czarkowski, Gina R Petroni, Mark Smolkin, William W Grosh, Kimberly A Chianese-Bullock, Donna H Deacon, Craig L Slingluff

**Affiliations:** 1 Department of Medicine/Division of Hematology-Oncology, University of Virginia, Charlottesville, VA 22908, USA; 2 Department of Surgery/Division of Surgical Oncology, University of Virginia, Charlottesville, VA 22908, USA; 3 Cancer Center, Charlottesville, VA 22908, USA; 4 Department of Public Health Sciences, University of Virginia Health System, Charlottesville, VA 22908, USA

**Keywords:** Immunotherapy, Human, Cytokines, T-Lymphocytes, Cytotoxic, Melanoma/im, Antigens, Neoplasm, Tumor vaccines

## Abstract

**Background:**

Cancers produce soluble and cell-associated molecules that can suppress or alter antitumor immunity. Preclinical studies suggest the disease burden may alter the cytokine profile of helper T cell responses to cancer antigens. We studied cytokine production by helper T cells responding to vaccination with 6 melanoma helper peptides (6MHP) in blood and lymph nodes.

**Methods:**

Twenty-three patients with stage IIIB-IV melanoma received a 6MHP vaccine. Antigen-reactive T cells from blood and draining lymph nodes were cultured, exposed to antigen, and then supernatants (days 2 and 5) were assayed for Th1 and Th2 cytokines. Results from 4 time points were compared to pre-vaccine levels.

**Results:**

Cytokine responses to vaccinating peptides were observed in 83% of patients. Th1 favoring responses were most common (17 of 19 responders). The most abundant cytokines produced were IFN-γ and IL-5 in the PBMC’s. IL-2 responses predominated in cells obtained from draining lymph nodes in 2-day culture but not in 5-day cultures. Patients with clinically measurable disease produced similar levels of total cytokine and similar degree of Th1 polarization as patients with no evidence of disease (NED).

**Conclusions:**

The MHC class II-associated peptides used in this study induced helper T cells with a Th1-biased cytokine response in both PBMC and sentinel immunized nodes. Most patients can mount a Th1 dominant response to these peptides. Future studies are needed to test newer vaccine adjuvants in combination with these peptides.

**Trial registration:**

CDR0000378171, Clinicaltrials: NCT00089219.

## Background

Most antigens selected for peptide-based cancer vaccines have been MHC class I restricted; they elicit predominantly CD8^+^ cytotoxic T-cell responses. The roles for MHC class II associated helper peptides and the CD4^+^ T-cells which they stimulate have often been viewed as secondary mechanisms in immunotherapy. Nevertheless, it is known that CD4^+^ T lymphocytes are important in generating effector T cell responses, in “licensing” dendritic cells, and in maintaining immunologic memory [1-4]. They mediate their effects, in part, by the cytokines they produce. The Th1 favoring cytokines (e.g.: IFN-γ, TNF-α, and IL-2) classically support cytotoxic T lymphocyte (CTL) responses, while the Th2 cytokines (e.g.: IL-4, IL-5, IL-10) support generation of antibody responses and may be antagonistic to induction of CTL responses.

We previously employed a tetanus helper peptide AQYIKANSKFIGITEL to stimulate helper T cells. The tetanus helper peptide has been immunogenic and induced Th1-predominant responses [5]. More recently, we have evaluated a multi-epitope vaccine incorporating 6 melanoma helper peptides (6MHP), restricted by HLA-DR molecules, that function as epitopes for CD4^+^ T cells. This 6MHP vaccine used in the current study is proven to be safe and shows some evidence of clinical activity as previously published [6]. Clinically, objective response was observed in 2 of 17 patients (12%) with measurable disease [6]. The vaccine induced antigen-specific CD4^+^ T cell responses in over 80% of patients. We previously showed that all 6 helper peptides used for this study are immunogenic and that MAGE-A3_281-295_ and tyrosinase_386-406_ have the most frequent responses [6,7]. Since, the cytokine profile of responding T cells is not previously reported we now report on cytokine profiles of helper peptide vaccinated patients.

Prior *in vitro* work with human peripheral blood mononuclear cells (PBMC) suggested that disease status (advanced measurable disease vs. clinically free of disease) may direct the Th1/Th2 dominance of T cell responses to helper peptides. In patients bearing measurable advanced renal cell cancer, T cell responses were generated *in vitro* with dendritic cells (DC) pulsed with helper peptides from MAGE-A6, and responses were highly Th2 dominant, manifested primarily by high IL-5 secretion [8]. In contrast, lymphocytes from patients rendered clinically free of disease by surgery had Th1-dominant responses to the same helper peptides, manifested by high secretion of IFN-γ [8]. The current report is intended to assess whether T cell responses to vaccination with 6 melanoma helper peptides (6MHP) are predominantly Th1 or Th2 dominant, and to obtain preliminary data on whether Th1/Th2 dominance varies as a function of clinical disease status.

## Results

Cytokine profiles from 23 vaccinated stage III and IV melanoma patients were assessed from PBMC’s and draining lymph nodes. The lymph nodes collected at day 22 of the 6-vaccine series were surgically resected from the draining nodal basins most proximal to the vaccine sites (sentinel immunized nodes, SIN). We assessed the Th1 favoring cytokines: IFN-γ, TNF-α and IL-2, and the Th2 favoring cytokines: IL-4, IL-5 and IL-10. There were three dose arms in the study, and there were no differences in toxicity nor response by dose (previously published) [6]. The cytokine profiling is performed on supernatants from cultures of lymphocytes derived from draining nodes and from peripheral blood.

### The Th1/Th2 cytokine profiles of T cell responses to helper peptide vaccination

**Figure 1:**
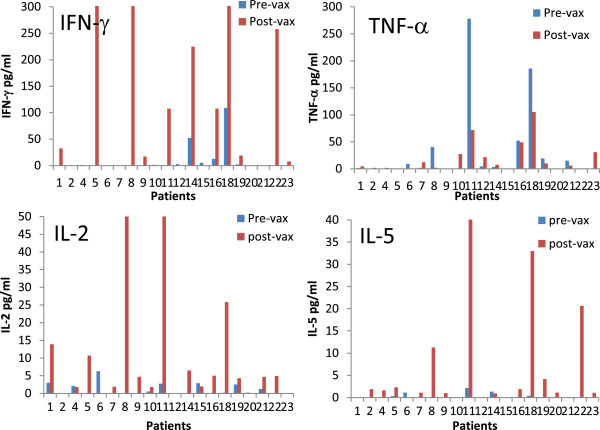
**Individual patient cytokine production pre and post vaccine.** The cytokine profiles for patients treated with 6 melanoma helper peptide vaccine. Cytokines were measured from PBMC’s cultured for 2 days with the 6 melanoma helper peptide mixture. Cytokines are shown in pg/ml. Arm A (patients 1-9) received 200 mcg, Arm B (patients 10-15) received 400 mcg and Arm C (patients 16-23) received 800 mcg. Cytokine cultures were performed in triplicate. All cytokine levels shown are corrected for background and negative control. Axes were scaled to show relative change from baseline. The ranges for the vertical differ between plots based on absolute cytokine level. IL-4 and IL-10 are available as Additional file 1: Figure S6.

Cytokines secreted by peripheral blood mononuclear cells (PBMC) and sentinel immunized node (SIN) lymphocytes were measured 2 days after pulsing them with the vaccinating 6 helper peptide pool. Th1 and Th2 cytokine production in response to the 6MHP pool is shown in Figure 1 for PBMC’s pre and post-vaccine. Cytokine levels post-vaccine were not different among dose arms of the study (all p values >0.05). An increase in total cytokine production by PBMC’s was observed in 19 of 23 patients (83%) from baseline (pre-vaccine) to post-vaccine, and responses greater than 4 times baseline were seen in 14 of 23 (61%). In terms of cytokine polarization for PBMCs post-vaccine, 17 of 19 responding patients had Th1 predominant responses; one patient had a Th2 predominant response.

The most abundant individual cytokine detected in response to helper peptide vaccination was IFN-γ, increasing from baseline of 8.7 to 121 pg/ml (Table 1) in PBMC’s. In terms of Th2 favoring cytokines, IL-5 was often induced by vaccination, but in relatively lower abundance than IFN-γ (post-vaccine mean of 20.7 pg/ml). Overall, IL-5 production increased in 14 of 23 patients. Changes in IL-4 and IL-10 were rarely significant and rarely changed above background controls. The values for SIN and additional PBMC values are listed in the tables. Values on the tables and figures reflect background adjustment and raw cytokines values were generally within the linear range of the cytokine bead assay (94%).

**Table 1:** Summary of 2-day response data

	**PBMC (n = 23)**			**SIN(n = 22)**
**Cytokine**	**Mean pre-vaccine(pg/ml)**	**Mean post-vaccine(pg/ml)**	**Number with increase(p value)**	**Mean at day 22**
IFN-γ	8.7	121	15 (p = 0.04)	51.2
IL-2	0.8	13.7	15 (p = 0.07)	62.3
TNF-α	15.2	16.1	10 (p = ns)	4.4
IL-4	0.8*	5.0*	7 (p = ns)	2.8
IL-5	0.2	20.7	14 (p = ns)	12.7
IL-10	1.1*	1.6*	9 (p = ns)	2.1
Th1 sum	24.8	150.9	17 (p = 0.036)	118
Th2 sum	2.1	27.3	14 (p = 0.042)	17.6
IFN vs IL-5	0.2 vs 8.7	20.7 vs 121	p = 0.005	p = 0.010
Th1 NED	12.1	174	p = 0.091	162
Th1 Disease	41.2	111		66
Total cytokines NED	14.3	211	p = 0.11	188
Total cytokines Disease	43.1	120		65

Means of cytokines and assessments of individual cytokine responses to vaccination with 6MHP. Increase from baseline required values within linear range of cytokine assay. Mean values reflect aggregate activity and outliers are notable as seen in the figures. All values are corrected for background PBMC is peripheral blood mononuclear cells and SIN are sentinel immunized nodes. NED = no evidence of disease.

*Some samples zeroed based on background correction.

**Figure 2:**
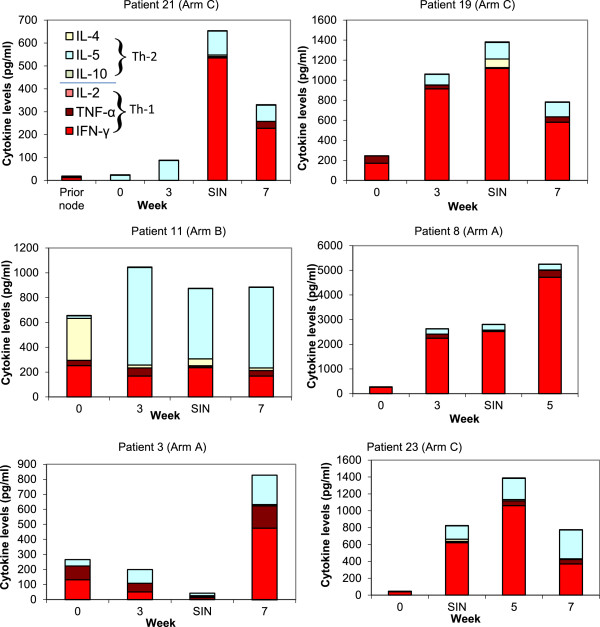
**Cytokine production at different time points following helper peptide vaccination.** Cytokine levels in 5-day supernatants are shown for 6 patients with proliferative responses to 6MHP vaccination whose PBMC were evaluated after stimulation with 6MHP. Th1 and Th2 cytokines are represented by stacked bars. The time in weeks after first vaccine is shown on the x-axis. The SIN was collected at day 22. The prior node for patient number 21 was collected prior to helper peptide vaccination and was analyzed with permission. It is included to illustrate the pre-vaccine cytokine milieu of the lymph nodes.

Cytokine profiles over time are shown for representative patients in Figure 2 (and Additional file 1). The figures demonstrate examples of enhanced cytokine production following helper peptide vaccination. It is evident that cytokine production fluctuates over time for certain cytokines. The primary discernible trend observed over 7 weeks is that patients tend to maintain their individual cytokine predominance, i.e. a Th1 predominant initial response tends to persist. Likewise, in the single patient with a Th2 predominant PBMC profile, that profile persisted throughout the observation period (patient 11, Figure 2).

### 5 Day lymphocyte culture increases overall cytokine production, but decreases IL-2 production

Cytokine production for 11 patients was analyzed by 5 day culture methodology (otherwise identical to 2-day culture). The overall magnitude of cytokine response was greater after 5 day culture for all patients (p = 0.003, Table 2), although variability is seen when examining individual cytokines. Individual cytokine levels were significantly greater on day 5 than day 2 for IFNγ, IL-2, and IL-5. The Th1 and Th2 cytokine levels are shown for each of those 11 patients in Additional file 1: Figure S1. Eight of the eleven patients had Th1 dominant responses post-vaccine, two had Th2 dominant responses (11, 18), and one had a balanced profile (22). The profiles in SIN-derived lymphocytes were similar, with 6 of 9 having Th1-dominant responses, one having a Th2 response and two having no response. In these 5 day supernatants from SIN derived lymphocytes, IFN-γ replaced IL-2 as the highest measured cytokine. The differences in cytokines between 2-day and 5-day cultures were evaluated as an exploratory endpoint, with significance levels shown in Table 2. Also notably, neither of the Th2 responders had received prior chemo.

**Table 2:** Comparison of 2 day to 5 day cytokine responses

	**2 Day(n = 23)**	**5 Day(n = 11)**	t-test*
IFN-γ	112	1380	**0.03**
TNF-α	17	92	0.45
IL-2	12	0.1	**0.004**
IL-10	1.6	12	0.062
IL-4	4.4	3.2	0.8
IL-5	20	267	**0.02**
Total	178	1756	**0.003**

Summary of cytokine values at 2-day culture versus 5-day culture following 6MHP vaccination. Mean cytokine at peak time point are shown in pg/ml and mean values are shown. Value are background and baseline corrected. Bold face highlights p-values <0.05. *The 5 day analysis represents a subset of the 2 day cohort so this is a descriptive analysis only.

### Clinical outcomes

**Figure 3:**
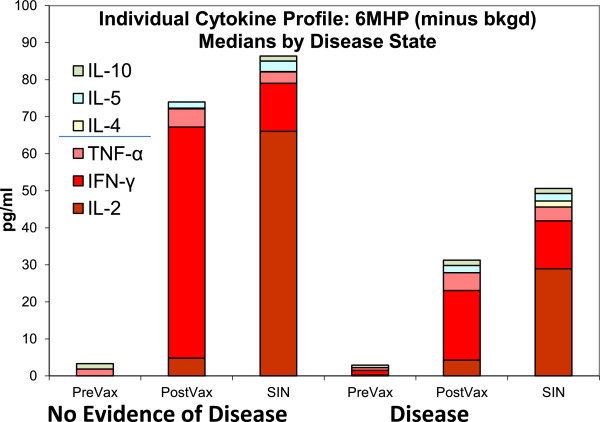
**Individual Cytokine Profiles; Medians by disease state.** Cytokine responses are compared by disease state where NED indicates no evidence of disease. Disease indicates unresectable metastatic melanoma. Median cytokine production is shown as stacked bars. SIN were collected at day 22. This is a 2-day culture assay where n = 23. All values are background corrected. Post-vaccine indicates the time point with highest magnitude of response and may differ for individual patients. This figure is shown to display relative proportions of individual cytokines, specifically the relative contribution of IL-2. There is no statistical difference between disease status groups overall.

The RECIST clinical response rate was 12% for the study [6]. Among the 23 patients evaluated for cytokine profiles, 11 had advanced metastatic disease, and 12 had no evidence of disease (NED) due to resected stage III or resected stage IV status. The IFN-γ change pre-vaccine to post-vaccine in the 2 day assay was 137 pg/ml in the NED group and 67 pg/ml in the metastatic group (p = 0.33, t-test). The IL-5 response was similar between groups (28.1 g/ml for NED and 5.5 pg/ml for metastatic, p = 0.93, t-test). No statistically significant difference in total cytokines production was observed as shown in Figure 3. Individually, one of 11 metastatic patients had a Th2-dominant response and no patients in the NED group had a Th2 response. Examination of the SIN cytokine production also failed to show a statistically significant difference in cytokine production by disease status (Table 1). Individually, one of 12 SIN in the NED group and none in the metastatic group had Th2-dominant responses.

In terms of survival, we observed that stage III and IV patients who were NED at the time of the vaccination achieved a mean survival of 4.9 years. The mean survival for patients with clinically measurable disease was 2.5 years. The 4 patients who had partial responses or stable disease by RECIST had nothing unique about their cytokine profiles at any of the 4 time points tested. They were all Th1 favoring responders by cytokine profiling. We previously reported that those 4 clinical responders all had proliferative responses to vaccine, delayed-typed hypersensitivity and autoimmune phenomena. Due to the small number of responders, we did not find significant correlations between any of the cytokines and clinical response.

### Th1/Th2 bias on a per-cell basis

**Figure 4:**
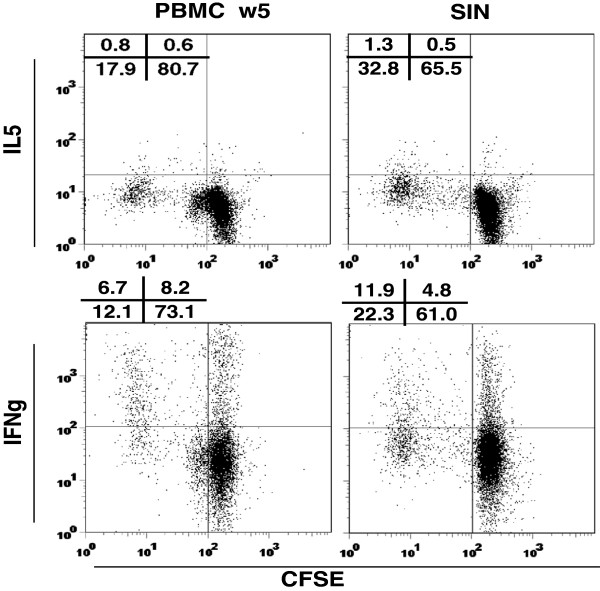
**Cytokine secretion in response to 6MHP stimulation.** PBMC (week 5, left panels) and SIN lymphocytes (day 22, right panels) were stimulated with 6MHP after CFSE labeling, then tested for IL-5 (top panels) production or IFN-γ (bottom panels) production. Data are shown for cells from a representative patient (patient 8) gated on lymphocytes, CD3-positive, and CD8-negative. The cells secreting cytokine are represented in the left upper quadrant of each plot, with percentages shown for each quadrant. IFN-γ producing cells are 8-9 fold more prevalent than IL-5 secreting cells in both PBMC and SIN’s.

Relative concentrations of Th1 and Th2 cytokines from bulk lymphocyte cultures may not accurately represent the Th1 or Th2 bias of responding lymphocytes. A measure with biologic relevance is the number of CD4^+^ cells secreting Th1 cytokines compared to the number secreting Th2 cytokines. Since IFN-γ and IL-5 were the predominant Th1 and Th2 cytokines in our assays, we measured those cytokines in helper T cells (CD8^neg^) proliferating 5 days in response to the 6MHP mixture for two representative patients by flow cytometry. CD4 can be downregulated after stimulation [9]; so positive selection of CD4^+^ T cells for this analysis could have been limited by downregulation of CD4 expression after antigen exposure. Thus, as a surrogate for proliferating CD4^+^ cells, we used proliferating CD3^+^CD8^neg^ cells defined by CFSE dilution. Among proliferating CD3^+^CD8^neg^ cells, IFNγ-secreting cells were 3- to 10-fold more prevalent than IL-5 secreting cells as measured by flow cytometric analysis (Figure 4). These findings correlated well with cytokine measurements in supernatants for the corresponding patients (patients 8 and 9). Among proliferating CD3^+^CD8^neg^ cells for patient 8, IL-5 was produced by only 4% of PBMC (collected week 5) and 4% of SIN lymphocytes, whereas IFN-γ was produced by 36% of PBMC and 35% of SIN lymphocytes (Figure 4). For a second patient (#9), IL-5 and IFNγ-secreting cells were 9% and 28% of CFSE-diluted cells, respectively, in PBMC (week 3) and 8% and 24% in the SIN (data not shown).

### Cytokine profiles in sentinel immunized nodes are not impacted by prior vaccination with class I associated peptides

**Figure 5:**
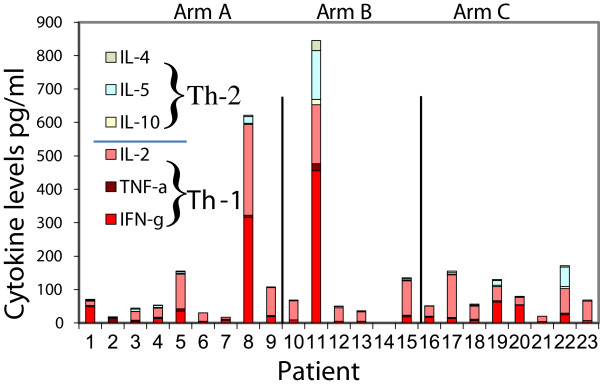
**Cytokine response in sentinel immunized lymph nodes.** Cytokines were measured from lymphocytes collected from sentinel immunized lymph nodes cultured for 2 days with the 6 melanoma helper peptide mixture. Arm A (patients 1-9) received 200 mcg, Arm B (patients 10-15) received 400 mcg and Arm C (patients 16-23) received 800 mcg. Cytokine cultures were performed in triplicate. All cytokine levels shown are corrected for background and negative control. The node was not able to be collected from patient #14.

**Figure 6:**
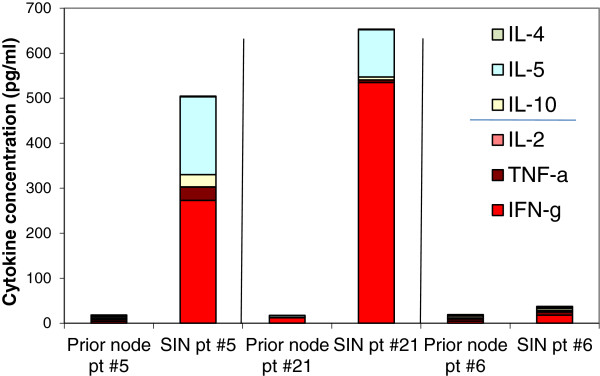
**Cytokine production from SIN lymphocytes in response to 6MHP stimulation in 3 patients with prior vaccinations.** The SIN responses for 3 patients from prior type I vaccine studies. SIN lymphocytes from pre-vaccine lymph nodes were cultured with the 6MHP, and supernatants were harvested at 40-48 h, and then evaluated with cytokine bead array (CBA) for levels of Th1 and Th2 cytokines. The SIN lymphocytes from the same patients were evaluated with cytokine bead array after 3 doses of 6MHP vaccination. Notably, prior vaccination with type I peptides does not seem to preclude a response to type II peptide vaccination.

A Th1 cytokine predominance was observed in 21 of 22 sentinel immunized nodes harvested at 3 weeks from the first vaccine (2-day assay, Figure 5). The most abundant individual cytokine in the SIN in 2-day supernatants, was IL-2, rather than IFN-γ, which was predominant from PBMC. Individually 17 lymph node samples had IL-2 as the predominant cytokine, while 3 produced more IFN-γ than IL-2 (and 2 were low producers). Due to the invasive nature of a lymph node biopsy, baseline nodes were not obtained for comparison as part of this trial, but 3 patients had had lymph nodes removed in other vaccine trials prior to this study. The three patients who had been on prior vaccine trials (Mel 36, 37, 39) [10-12] did not receive prior melanoma helper peptides. For these patients, we evaluated the response of SIN lymphocytes to the 6MHP, compared to SIN data from the present trial. In response to stimulation with the 6MHP, there was no detectable cytokine response in the pre-Mel41 lymph node cells (Figure 6). After 3 weeks of vaccination with 6MHP, proliferative responses to the 6MHP were observed [6], and strong cytokine responses were detected for two of the three patients (Figure 6).

## Discussion

Helper peptides for cancer vaccination have previously been shown to be immunologically important both alone and as adjuvants [5,13]. We previously observed that other helper or class II peptides evoked Th1 dominant cytokine responses from stimulated lymphocytes [12] and now hypothesize that helper peptides specifically from melanoma antigens would also produce Th1 favoring responses. Further, based on published work [8], we hypothesized that patients with measurable disease might display more Th2-biased responses.

The cytokine profiles of T cells induced by the helper peptides in this vaccine are most consistent with a Th1 dominant profile in 14 of 19 responders (74%) of responders. Interestingly, 2 patients had a Th2-dominant response (one for the 2-day assay and one for the 5-day). Balanced Th1/Th2 responses occurred in 3 patients. Th-1 responses are believed to be most supportive of phagocytic and cytotoxic type tumor responses; the induction of Th2 dominant responses in two patients may be antagonistic to the induction of protective immunity.

It is not known whether the observed Th2 responses represent induction of new Th2 responses, a selective depletion of Th1-responding cells, or a suboptimal vaccination strategy. Future studies to identify molecular mediators of Th2 responses and to characterize further the pleiotropic effects of these cytokines may be informative. Likewise, future evaluation of Th-17 responses elicited by helper peptides is also needed and may further elucidate the relative contribution of Th-2 cytokines in helper responses. In this study, most patients maintained a Th1 dominant response throughout the course of vaccine therapy (Figure 5). Analysis of delayed type hypersensitivity data [6] also supports an immune response up to 9 months. Questions remain about the need for booster vaccines; these data do not rule out the possibility that booster vaccines may favorably modify the helper T cell response.

The total cytokine production significantly increased from 2 day to 5 day culture, which is not surprising; however, high IL-2 levels were seen in draining nodes in 2 day cultures which were not seen in 5 day node cultures. This likely represents consumption of IL-2 by T cells, during longer culture and could apply either to expansion of activated T cells or induction of regulatory T cells, both of which express the high affinity IL-2 receptor alpha chain (CD25) [14]. Central memory T cells (T_CM_) respond to antigen by high IL-2 production [15], thus, the disparate cytokine profiles in the SIN and PBMC suggest that antigen reactive cells in the node (but not in the PBMC) may be enriched for T_CM_. The loss of IL-2 dominance as T_CM_ respond to antigen and proliferate is also consistent with that observation.

While nearly all patients maintained their overall cytokine profile over time, in a few cases, the Th2/Th1 ratio increased in culture over time, toward slightly lower Th1 predominance. These anecdotal cases highlight the relevance of following immunologic endpoints over time and also highlight the wide degree of variability seen when dealing with human immune monitoring.

It has been reported that PBMC from patients with advanced measurable malignancy may have Th2-dominant responses to helper peptides, whereas those rendered clinically free of disease may have Th1-dominant responses [8]. Our study differs from that report in part by being in melanoma patients rather than renal cell cancer, but more importantly because the induction of the T cell response was in vivo (tested in vitro) rather than being induced in vitro. Our findings do show non-significant trends toward that prior observation, since the only Th2-dominant responses we observed in either 2 or 5 day assay were in metastatic patients with measurable disease, and all of the 12 NED patients had a Th1-dominant response. Also, the total cytokine produced in NED patients trended higher than among those with measurable disease. However, these were not significant differences and were much less pronounced than the prior data after in vitro sensitization. Overall, Th1-dominant responses were most common among both subsets of patients. Anecdotally, prior chemotherapy exposure was not associated with either of two Th2 responses observed.

The observed cytokine response rate of 83% mirrors the proliferative response rate previously reported (81%), but neither immune assay correlates well with the clinical response rate of 12%. The median overall survival for stage IV patients in this study was 2.5 years which is very favorable, but may be influenced by referral bias. In another study with this 6MHP vaccine, immune responses to these 6MHP were strongly and significantly associated with overall survival [13]. However, helper peptide vaccination remains experimental. Further work is needed to optimize adjuvants and to identify measures of immune response associated with favorable outcome. Since this study was designed, we have found that GM-CSF decreases both CD4+ and CD8+ responses to melanoma vaccines in incomplete Freund’s Adjuvant [16]; thus, we anticipate that vaccination with melanoma helper peptides without GM-CSF or in different adjuvant systems may be even more effective at inducing high magnitude Th1-dominant immune responses.

## Conclusions

The MHC class II-associated peptides used in this study induced helper T cells with a Th1-biased cytokine response in both PBMC and sentinel immunized nodes. IL-2 was the dominant Th1 cytokine produced early from SIN, but not from PBMC, and this is consistent with a central memory response in the SIN, which is not surprising since T_CM_ are expected to reside in lymphoid organs. There were exceptions in that several patients had cytokine responses that were either Th2-dominant or Th1-Th2 balanced. The analysis of cytokine profiles over time also suggests that induced helper T cells respond in a specific and stable manner when re-exposed to vaccinating peptides. Clinical disease status did not bias the cytokine polarization in a significant way, though the overall magnitude of cytokine production trends higher in patients without clinically evident disease. Studies in larger patient populations may find significant differences that were not evident here, and if so, that could support different immunotherapy approaches depending on the clinical disease status. Thus, as vaccine adjuvants are improved, analysis of the effects on cytokine profiles in blood and nodes may help to identify patients who may benefit most from helper peptide vaccines.

## Methods

### Patients

Patients with AJCC stage IIIB, IIIC or IV melanoma with or without measurable disease were eligible for this vaccine trial. Candidates were required to express at least one of the 5 HLA-DR alleles by which CD4 T cell recognition for these 6 peptides had been defined: HLA-DR1, DR4, DR11, DR13, DR15. Other inclusion criteria and details of the study have been reported [6]. Patients were studied following informed consent, and with Institutional Review Board (HIC#10464) and FDA approval (BB-IND #10825). Baseline demographics are described in Additional file 1: Table S3.

### Vaccine composition

All patients received a vaccine comprising 6 melanoma peptides reported to be restricted by one or more HLA-DR molecules. The peptides and their reported HLA-DR restrictions are AQNILLSNAPLGPQFP (Tyrosinase_56-70_, HLA-DR4) [16], FLLHHAFVDSIFEQWLQRHRP (Tyrosinase_386-406_, HLA-DR15) [17], RNGYRALMDKSLHVGTQCALTRR (Melan-A/MART-1_51-73_, HLA-DR4) [18], TSYVKVLHHMVKISG (MAGE-3_281-295_, HLA-DR11) [19], LLKYRAREPVTKAE (MAGE-1,2,3,6_121-134_, HLA-DR13) [20], and WNRQLYPEWTEAQRLD (gp100_44-59_, HLA-DR4 & -DR1) [21,22]. An alanine residue has been added at the N-terminus of AQNILLSNAPLGPQFP (Tyrosinase_56-70_), to prevent cyclization of the N terminal glutamine residue in the originally described sequence QNILLSNAPLGPQFP [16,23]. They were prepared under good manufacturing practice (GMP) conditions as described [6].

The peptides were administered with 110 mcg GM-CSF (Berlex, Seattle, WA) in a stable emulsion with 1 ml Montanide ISA-51 adjuvant (Seppic, Inc., Paris, France/ Fairfield, NJ) days 1, 8, 15, 29, 36, and 43 (weeks 0, 1, 2, 4, 5, and 6). The first 3 vaccinations were divided between two injection sites (primary and replicate), and the last 3 vaccinations were delivered to the primary injection site only. At each injection site, half was administered subcutaneously and half was administered intradermally. Vaccine was administered at 3 dose levels (200 ug, 400 ug and 800 ug, arms A, B and C) with no differences in AE’s nor responses between the dose levels (previously reported). In addition to peptides used in the vaccine, a tetanus helper peptide AQYIKANSKFIGITEL and the irrelevant peptide from HIV gag protein, SLYNTVATL were used in laboratory analyses [11,24].

### Collection of peripheral blood mononuclear cells (PBMC)

Blood was drawn weeks 0, 1, 3, 5, 7, 12, and 18, and at months 6, 9, 12, 18, and 24. Those drawn on weeks 1, 3, 5, and 7 correspond to one week after vaccines 1, 3, 4, and 6, respectively. Lymphocytes isolated from peripheral blood by Ficoll gradient centrifugation were cryopreserved in 10% DMSO/90% serum. Several vaccinated patients were unable to complete all blood draws or had non-viable lymphocytes at time of cytokine analysis; 23 of 37 patients had a minimum of 3 time points with adequate and viable lymphocytes.

### Harvest of the sentinel immunized node (SIN)

On day 22 (week 3), the lymph node draining the replicate immunization site, the sentinel immunized node (SIN), was localized and harvested under local anesthesia as reported [25,26]. A central slice of the SIN was preserved in formalin, and the remainder was dissociated mechanically into a single cell suspension of lymphocytes, and cryopreserved by the University of Virginia’s Tissue Procurement Facility.

### Proliferation and cytokine assays

Responses to the six melanoma helper peptides were assessed *in vitro* by measuring proliferation after antigen exposure. PBMC were thawed in RPMI 1640 medium with 5% heat inactivated human AB serum (HuAB; Gemini Bio-Products, West Sacramento, CA) and DNAse (Worthington Biochemical Corporation, Lakewood, New Jersey, Cat # LS002139; 100 mg, 2,430 units/mg dry weight) at 100 U/ml and washed twice. Cells were adjusted to 1×10^6^ cells/ml in tissue culture medium consisting of AIM V (Invitrogen Corporation/GIBCO, Carlsbad, CA) with heat inactivated 10% HuAB serum, plus each of the following 11 conditions: 1) media only; 2) bovine serum albumin (BSA; Sigma-Aldrich, St. Louis, MO; 10 ug/ml); 3) tetanus peptide (10 mcg/ml); 4-9) each of the 6 melanoma helper peptides (10 mcg/ml, individually tested); 10) 6 melanoma helper pool (6MHP; all 6 peptides at 10 mcg/ml per peptide); and 11) PHA (Sigma; at 5 mcg/ml). PBMC from two normal donors (Virginia Blood Services, Charlottesville, VA) were included as controls and were stimulated with each of the following 13 conditions: 1) media only; 2) BSA; 3) Tetanus peptide; 4-9) 6-melanoma peptide pool; 10) PHA; 11) influenza lysate, 12) lysate control (Microbix Biosystems Inc., Toronto, Ontario); or 13) PHA. Cultures were assayed in triplicate in 96-well flat bottom cluster dishes and were incubated 5d at 37°C and 5% CO_2_.

### Cytokine assays

On day 2 or day 5 of the 5-day proliferation assay cultures described above, 50 μl supernatant was removed from each well of the triplicate cultures, pooled and stored at -80°C until tested for Th1 (IFN-gamma, IL-2, TNF-alpha) and Th2 (IL-4, IL-5, IL-10) cytokines by cytokine bead array (BD Biosciences, Franklin Lakes, NJ).

### CFSE labeling and in vitro stimulation

Cryopreserved PBMC were thawed in pre-warmed medium (RPMI 1640 + 5% fetal calf serum) containing DNase (Worthington Biochemical Corp., Lakewood, NJ) at 100 units/ml. Viability was determined by Trypan blue dye exclusion, and viable cells were adjusted to 2-4 million/ml in 0.2% BSA in PBS. Cells were labeled using the Vybrant CFDA SE Cell Tracer kit (Invitrogen Corporation/Molecular Probes). Carboxy-fluorescein diacetate, succinimidyl ester (CFDA SE; referred to hereafter as CFSE) was dissolved in DMSO to 10 mM. and diluted to 1 μM in PBS. Equal volumes of cells and dye were combined, incubated 10 minutes at 37°C then washed in AIM V (Gibco/Invitrogen) supplemented with heat inactivated 5% HuAB serum. After stimulation 5d at 37°C with peptide (10 ug/ml) or CD3/CD28 beads (Miltenyi Biotec, Inc., Auburn, CA), cells were collected and prepared for flow cytometric analysis.

### Immunological reagents for flow cytometry

Fluorescent tagged antibodies used were specific for: CD3 allophycocyanin (APC), CD3 APC-cyanine7 (Cy7), CD4 phycoerythrin (PE), IL5 PE (BD Biosciences, San Jose CA); CD8 PE-Cy7 (Beckman Coulter Miami FL); IFN-gamma APC, FoxP3 (clone PCH101) and its isotype control (eBiosciences, San Diego CA); CD25-PE (Miltenyi Biotec Inc, Auburn CA).

### Intracellular detection of IFNγ and IL-5

Intracellular cytokine (ICC) production by proliferating cells was determined on peptide-stimulated CFSE-labeled PBMC after phorbol 12-myristate 13-acetate (PMA; 40 ng/ml; Sigma-Aldrich) plus ionomycin (1 μM; Sigma). Brefeldin A (10 μg/ml; Sigma-Aldrich) was added to cultures after one hour. After stimulation of PBMC with the pool of 6MHP for 5 days, PMA and Ionomycin in the presence of Brefeldin A was added for 5 additional hours detect cytokines produced by dividing cells. For flow cytometric analysis, the CD3posCD8neg T cell population served as a surrogate CD4 population because PMA is known to down-modulate surface CD4. Cells were harvested 5h later and surface-stained with anti–CD3 APC-Cy7 and –CD8-PE-Cy7, then washed, fixed and permeabilized (Cytofix/CytoPerm™, BD Biosciences) before staining with anti-IL5 PE and anti-IFN-gamma APC. Cells were collected on a modified Becton Dickinson FACSCalibur dual laser (488 nm and 637 nm) benchtop cytometer (FACSCalibur A) capable of 7 parameter (five color) analysis. Data were analyzed with FloJo (Treestar, Ashland OR) software. Gates and parameter thresholds were set using unstained cells as controls.

### Statistical methods

Positive and negative controls were performed and all results adjusted accordingly. Non-specific and background cytokine production was accounted for by use of controls and baseline subtraction. Cytokine levels were rescaled using the square root transformation for some figures, and in the case of PBMC’s, readjusted by subtraction of the baseline cytokine level. Summary statistics were calculated for levels of cytokine. Paired and two sample t-tests were used to compare cytokine levels across various factors. Levels of cytokine were assessed across treatment arms using linear regression methods. Survival function was performed in SAS. The overall study was not powered for assessing multiple cytokine profiles, however cytokine profiling by study arm and disease status were pre-planned analyses in the study protocol.

## Competing interests

The author’s declare that they have no competing interests.

## Authors’ contributions

CLS and WCO conceived and designed the study and experiments. WCO, DD and AZ completed the cytokine bead arrays and flow cytometry. CLS and WWG recruited patients. MS and GRP oversaw statistical analysis and data safety during the trial. WWG and CLS enrolled patients on the clinical trial PMD, CLS and WCO wrote and revised the manuscript. PMD, CLS, AZ and WCO performed data analysis and developed figures. All authors read and approved the final manuscript.

## Supplementary Material

Additional file 1: Table S1 Peptides and parent proteins in this study. **Table S2.** Updated patient survival table (as of 3/14/2014). **Table S3.** HLA-DR distribution. **Figure S1.** This figure shows the cytokine response to 6MHP vaccination upon 5-day culture. **Figure S2.** This figure demonstrates the breadth of cytokine response to for a single patient to each of the relevant helper peptides. **Figure S3.** This figure is a tally of the sum of the means of Th1 and Th2 favoring cytokines measured in PBMC at their highest post-vaccine time point. **Figure S4.** Day 2 and 5 Interferon-γ Responses to 6MHP, means by arm. **Figure S5.** Flow cytometry experiment demonstrating change in CD4+, CD25^hi^, FoxP3+ regulatory T cells following helper peptide vaccination. **Figure S6.** These figures are the additional cytokines not shown in figure #1. **Figure S7.** This figure provides additional plots to figure 2. **Table S4.** sample data table illustrating patient variability. Click here for file
