# Supplementary material for: A melanoma helper peptide vaccine increases Th1 cytokine production by leukocytes in peripheral blood and immunized lymph nodes
Source: J Immunother Cancer. 2014 Jul 15;2:23. doi: 10.1186/2051-1426-2-23 (PMC4131803; doi:10.1186/2051-1426-2-23)
Supplement: Additional file 1: Table S1 — Peptides and parent proteins in this study. Table S2. Updated patient survival table (as of 3/14/2014). Table S3. HLA-DR distribution. Figure S1. This figure shows the cytokine response to 6MHP vaccination upon 5-day culture. Figure S2. This figure demonstrates the breadth of cytokine response to for a single patient to each of the relevant helper peptides. Figure S3. This figure is a tally of the sum of the means of Th1 and Th2 favoring cytokines measured in PBMC at their highest post-vaccine time point. Figure S4. Day 2 and 5 Interferon-γ Responses to 6MHP, means by arm. Figure S5. Flow cytometry experiment demonstrating change in CD4+, CD25hi, FoxP3+ regulatory T cells following helper peptide vaccination. Figure S6. These figures are the additional cytokines not shown in figure #1. Figure S7. This figure provides additional plots to figure 2. Table S4. sample data table illustrating patient variability. [file 2051-1426-2-23-S1.docx]

**Supplemental Figures:**

**Supplemental Table 1: Peptides and parent proteins in this study**

| **Epitope** | **Sequence** | **Allele** | **Reference** |
| --- | --- | --- | --- |
| **Tyrosinase 56-70** | **(A)QN ILLSNAPLGPQFP** | **HLA-DR4** | Topalian 1996 |
| **Tyrosinase 386-406** | **FLL HHAFVDSIFEQWLQRHRP** | **HLA-DR15** | Kobayashi 1998 |
| **Melan-A/MART-1 51-73** | **RNG YRALMDKSLHVGTQCALTRR** | **HLA-DR4** | Zarour 2000 |
| **MAGE-3 281-295** | **TSY VKVLHHMVKISG** | **HLA-DR11** | Manici 1999 |
| **MAGE-1,2,3,6 121-134** | **LLK YRAREPVTKAE** | **HLA-DR13** | Chaux 1999 |
| **gp100 44-59** | **WNR QLYPEWTEAQRLD** | **HLA-DR4 & -DR1** | Halder 97/ Li 1998 |

**Supplemental Table 2: Updated patient survival table (as of 3/14/2014)**

| **No Evidence of disease** | | **Measurable Disease** | |
| --- | --- | --- | --- |
| **Patient #** | **Years** | **Patient #** | **Years** |
| 564 | 1.1 | 504 | 7.0 |
| 636 | 1.6 | 352 | 1.2 |
| 740 | 3.6 | 335 | 1.2 |
| 784 | 0.8 | 537 | 3.0 |
| 847 | 0.5 | 550 | 9.5 |
| 819 | 4.0 | 582 | 1.5 |
| 872 | 0.3 | 625 | 0.3 |
| 425 | 6.1 | 589 | 3.0 |
| 485 | 8.5 | 635 | 1.4 |
| 842 | 3.0 | 685 | 0.3 |
| 701 | 2.5 | 681 | 0.6 |
| 677 | 7.0 | 683 | 5.7 |
| 699 | 2.0 | 739 | 5.0 |
| 727 | 7.8 | 749 | 0.2 |
| 729 | 6.6 | 743 | 0.4 |
| 381 | 6.7 | 647 | 0.7 |
| 216 | 3.1 | 719 | 3.2 |
| 827 | 5.5 | 797 | 0.7 |
| 871 | 3.6 | 621 | 1.9 |
|  |  | 576 | 2.1 |

**Supplemental Table 3:** HLA-DR distribution

**Supplemental Figure 1**

This figure shows the cytokine response to 6MHP vaccination upon 5-day culture. Individual cytokines are stacked for comparison. Values are adjusted for background and negative control. In figure B the same assay is shown for the lymphocytes collected from the sentinel immunized lymph node. Patients 2 and 18 had non-viable nodal lymphocytes and are not shown.

**A**

**B**

**Supplemental figure 2:** This figure demonstrates the breadth of cytokine response to for a single patient to each of the relevant helper peptides. Th1 favoring cytokines (IFN-γ, IL-2 and TNF-α) are shown above the x-axis and Th2-favoring cytokines (IL-4, IL-5, and IL-10) are shown below the x-axis for comparison purpose. 6MHP bars are the cytokine responses to stimulation with the 6 melanoma helper peptide pool. TSY is response to the MAGE A3. WNR is response to Gp100. AQN and FLL are the two tyrosinase derived helper peptides. The patient shown in panel A is patient number 17 and B is patient 8. The numbers in parentheses are the weeks since vaccination.

**A**

**B**

**Supplemental Figure 3:** This figure is a tally of the sum of the means of Th1 and Th2 favoring cytokines measured in PBMC at their highest post-vaccine time point. NED = no evidence of disease (ie resected stage III or resected stage IV patients). Dz = clinical evidence of disease (ie stage IV patients). P values are 0.091 and 0.11 for the comparisons shown.

**Supplemental Figure 4: Day 2 and 5 Interferon-γ Responses to 6MHP, means by arm.** This figure primarily shows that lack of difference between dose arms of the vaccine. Also shown are differences between 2-day and 5-day assays. The IFN-γ profiles of PBMC’s and sentinel nodes stimulated with 6MHP 2 days and 5 days after pulsing with 6MHP. IFN-γ cytokine responses by study arm for the three vaccine dose levels (arm A = 200mcg, arm B =400mcg, arm C =800mcg). IFN-γ is shown since it was the most predominant cytokine, but the other 5 cytokines tested had similar degrees of inter-patient variability and none had discernable differences across dose arms of the study. Values for these plots were square rooted to allow display on a single plot. Arm B, Day-5 represents a single patient, so no variation measure is shown.

A

B

C

A

B

C

A

B

C

A

B

C

0

100

400

900

1600

Day 2

Day 5

Day 5

Day 2

**IFN-γ production (pg/mL)**

PBMC’s

Sentinel immunized nodes

Supplemental Figure 5: Flow cytometry experiment demonstrating change in CD4+, CD25^hi^, FoxP3+ regulatory T cells following helper peptide vaccination. Lymphocytes were collected from both peripheral blood and sentinel immunized node and assayed without stimulation.


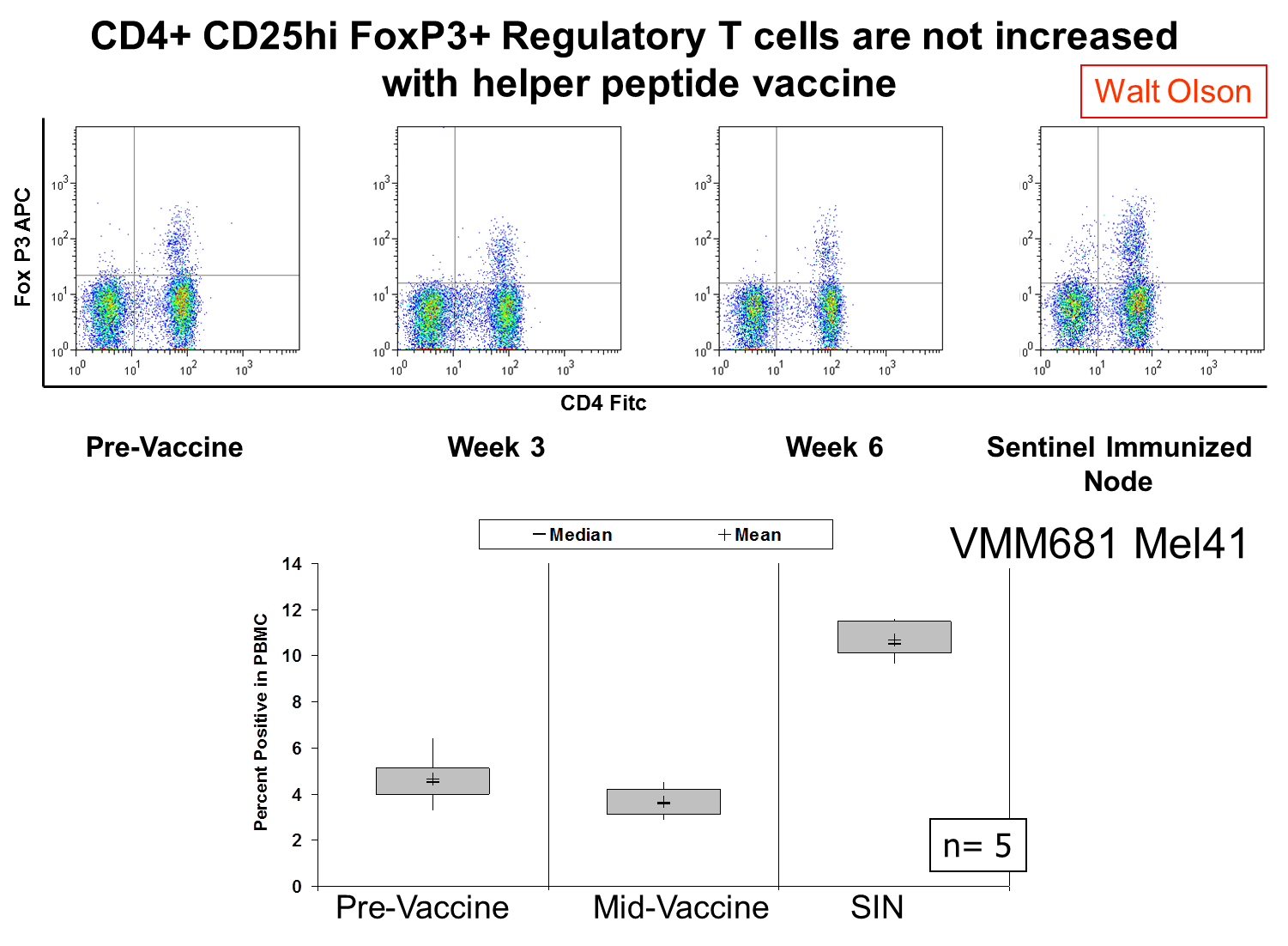


**Supplemental Figure 6:** These figures are the additional cytokines not shown in figure #1. IL-4 and IL-10 PBMC cytokine responses in 2-day culture following stimulation wit 6MHP pool. The only response observed was in patient 11 (the single Th2 responder in the 2-day assay)

**Supplemental Figure 7:** This figure provides additional plots to main figure 3. These are individual patient cytokine responses at varying time points for additional patients not able to be show in the body of the paper.

VMM729

VMM 819

VMM 381

VMM 216

**Supplemental Table 4:** This sample data table is included to illustrate the individual patient variability; it is not inclusive. For brevity, the baseline values, replicates, and additional time points are excluded. Individual peptides, proliferation data and day 5 data are excluded, but available upon request. M = metastatic, PD= progressive disease, NED = no evidence of disease, PR = partial response, SD = stable disease.

|  |  |  |  | **Sample pg/ml** | | | | | | | |  | |  | |  |
| --- | --- | --- | --- | --- | --- | --- | --- | --- | --- | --- | --- | --- | --- | --- | --- | --- |
|  | **Treatment** | **Arm** | **Disease** | **IFN-g** | **TNF-a** | **IL-10** | **IL-5** | **IL-4** | **IL-2** | **Sum of TH1 Cytokines** | **Sum of Th2 Cytokines** | | **Total Cytokines** | |  |  |
| 1 | PreVax | 1 | M→PD | 0.0 | 1.6 | 0.0 | 0.0 | 0.0 | 3.0 | 4.6 | 0.0 | | 4.6 | |  |  |
| 2 | PreVax | 1 | M→PD | 0.0 | 0.0 | 0.0 | 0.0 | 0.0 | 0.0 | 0.0 | 0.0 | | 0.0 | |  |  |
| 3 | PreVax | 1 | M→SD | 1.2 | 0.0 | 0.0 | 0.0 | 0.0 | 2.1 | 3.3 | 0.0 | | 3.3 | |  |  |
| 4 | PreVax | 1 | NED | 0.0 | 0.0 | 1.0 | 0.3 | 0.2 | 0.0 | 0.0 | 1.5 | | 1.5 | |  |  |
| 5 | PreVax | 1 | NED | 0.0 | 9.1 | 3.0 | 1.1 | 0.0 | 6.3 | 15.4 | 4.1 | | 19.4 | |  |  |
| 6 | PreVax | 1 | NED | 0.0 | 1.9 | 0.0 | 0.0 | 0.0 | 0.0 | 1.9 | 0.0 | | 1.9 | |  |  |
| 7 | PreVax | 1 | NED | 0.0 | 40.3 | 2.2 | 0.0 | 0.1 | 0.0 | 40.3 | 2.4 | | 42.8 | |  |  |
| 8 | PreVax | 1 | NED | 0.0 | 0.0 | 1.4 | 0.0 | 1.3 | 0.0 | 0.0 | 2.7 | | 2.7 | |  |  |
| 10 | PreVax | 2 | M→PD | 1.4 | 0.0 | 0.0 | 0.0 | 0.0 | 0.5 | 1.9 | 0.0 | | 1.9 | |  |  |
| 11 | PreVax | 2 | NED | 0.0 | 278.3 | 81.8 | 2.1 | 11.9 | 2.7 | 281.1 | 95.8 | | 376.9 | |  |  |
| 12 | PreVax | 2 | NED | 3.0 | 4.8 | 4.1 | 0.0 | 0.0 | 0.0 | 7.8 | 4.1 | | 11.8 | |  |  |
| 13 | PreVax | 2 | NED | 52.2 | 3.2 | 2.5 | 1.3 | 0.0 | 0.0 | 55.4 | 3.8 | | 59.2 | |  |  |
| 14 | PreVax | 2 | NED | 5.5 | 0.0 | 0.0 | 0.0 | 1.9 | 2.9 | 8.4 | 1.9 | | 10.3 | |  |  |
| 16 | PreVax | 3 | M→PD | 12.6 | 52.1 | 0.2 | 0.1 | 2.3 | 0.1 | 64.7 | 2.6 | | 67.4 | |  |  |
| 17 | PreVax | 3 | M→PD | 108.7 | 185.7 | 0.0 | 0.4 | 8.4 | 0.2 | 294.6 | 8.8 | | 303.4 | |  |  |
| 18 | PreVax | 3 | M→PR | 2.5 | 19.2 | 3.9 | 0.0 | 1.3 | 2.5 | 24.2 | 5.2 | | 29.3 | |  |  |
| 19 | PreVax | 3 | M→SD | 0.0 | 0.0 | 0.0 | 0.0 | 0.0 | 0.2 | 0.2 | 0.0 | | 0.2 | |  |  |
| 20 | PreVax | 3 | NED | 0.0 | 15.1 | 0.0 | 0.0 | 0.0 | 1.2 | 16.3 | 0.0 | | 16.3 | |  |  |
| 21 | PreVax | 3 | NED | 0.0 | 0.0 | 7.0 | 0.0 | 0.0 | 0.0 | 0.0 | 7.0 | | 7.0 | |  |  |
| 22 | PreVax | 3 | NED | 0.0 | 0.0 | 0.0 | 0.0 | 0.0 | 0.0 | 0.0 | 0.0 | | 0.0 | |  |  |
| 1 | PostVax | 1 | M→PD | 32.6 | 4.8 | 1.6 | 0.0 | 0.0 | 13.9 | 51.3 | 1.6 | | 52.9 | |  |  |
| 2 | PostVax | 1 | M→PD | 6.5 | 1.7 | 1.9 | 1.9 | 0.0 | 0.4 | 8.5 | 3.8 | | 12.3 | |  |  |
| 3 | PostVax | 1 | M→SD | 0.0 | 1.7 | 0.0 | 1.6 | 0.0 | 1.8 | 3.5 | 1.6 | | 5.1 | |  |  |
| 4 | PostVax | 1 | NED | 525.2 | 0.0 | 6.4 | 2.3 | 1.1 | 10.7 | 535.9 | 9.7 | | 545.6 | |  |  |
| 5 | PostVax | 1 | NED | 0.0 | 0.0 | 0.0 | 0.0 | 1.3 | 0.0 | 0.0 | 1.3 | | 1.3 | |  |  |
| 6 | PostVax | 1 | NED | 0.4 | 12.2 | 0.0 | 1.1 | 0.0 | 1.9 | 14.6 | 1.1 | | 15.6 | |  |  |
| 7 | PostVax | 1 | NED | 319.8 | 2.1 | 3.7 | 11.3 | 0.9 | 51.4 | 373.3 | 15.9 | | 389.2 | |  |  |
| 8 | PostVax | 1 | NED | 17.1 | 0.0 | 0.0 | 1.0 | 0.0 | 4.7 | 21.7 | 1.0 | | 22.7 | |  |  |
| 10 | PostVax | 2 | M→PD | 0.0 | 27.5 | 1.5 | 0.0 | 0.0 | 1.8 | 29.3 | 1.5 | | 30.7 | |  |  |
| 11 | PostVax | 2 | NED | 107.8 | 71.9 | 3.7 | 141.1 | 37.4 | 95.2 | 274.8 | 182.2 | | 457.0 | |  |  |
| 12 | PostVax | 2 | NED | 0.0 | 21.4 | 3.2 | 0.0 | 0.0 | 0.0 | 21.4 | 3.2 | | 24.7 | |  |  |
| 13 | PostVax | 2 | NED | 224.8 | 7.5 | 0.0 | 0.9 | 0.0 | 6.5 | 238.7 | 0.9 | | 239.7 | |  |  |
| 14 | PostVax | 2 | NED | 0.0 | 0.0 | 0.0 | 0.0 | 0.0 | 2.0 | 2.0 | 0.0 | | 2.0 | |  |  |
| 16 | PostVax | 3 | M→PD | 107.7 | 49.1 | 0.0 | 1.9 | 2.5 | 5.0 | 161.9 | 4.5 | | 166.3 | |  |  |
| 17 | PostVax | 3 | M→PD | 505.0 | 105.4 | 0.0 | 33.0 | 14.2 | 25.8 | 636.1 | 47.2 | | 683.4 | |  |  |
| 18 | PostVax | 3 | M→PR | 18.8 | 9.9 | 4.7 | 4.2 | 1.6 | 4.3 | 33.0 | 10.5 | | 43.4 | |  |  |
| 19 | PostVax | 3 | M→SD | 0.6 | 0.0 | 0.6 | 1.1 | 0.0 | 0.0 | 0.6 | 1.7 | | 2.3 | |  |  |
| 20 | PostVax | 3 | NED | 0.0 | 6.0 | 0.7 | 0.0 | 0.0 | 4.7 | 10.7 | 0.7 | | 11.4 | |  |  |
| 21 | PostVax | 3 | NED | 258.0 | 0.0 | 0.0 | 20.6 | 0.0 | 4.9 | 262.9 | 20.6 | | 283.5 | |  |  |
| 22 | PostVax | 3 | NED | 7.8 | 30.7 | 0.0 | 1.1 | 0.3 | 0.0 | 38.5 | 1.4 | | 39.9 | |  |  |
| 1 | SIN | 1 | M→PD | 48.7 | 3.7 | 1.6 | 1.9 | 1.8 | 13.1 | 65.6 | 5.3 | | 70.9 | |  |  |
| 2 | SIN | 1 | M→PD | 0.0 | 14.1 | 0.0 | 1.3 | 1.5 | 1.3 | 15.4 | 2.8 | | 18.1 | |  |  |
| 3 | SIN | 1 | M→PD | 3.7 | 4.6 | 0.4 | 7.3 | 2.6 | 25.7 | 33.9 | 10.3 | | 44.3 | |  |  |
| 4 | SIN | 1 | M→SD | 13.2 | 3.7 | 2.3 | 5.4 | 0.8 | 27.6 | 44.5 | 8.5 | | 53.0 | |  |  |
| 5 | SIN | 1 | NED | 35.5 | 6.8 | 1.6 | 4.5 | 2.0 | 104.2 | 146.6 | 8.1 | | 154.7 | |  |  |
| 5 | SIN | 1 | NED | 4.3 | 0.8 | 0.0 | 0.0 | 0.0 | 24.8 | 30.0 | 0.0 | | 30.0 | |  |  |
| 6 | SIN | 1 | NED | 8.1 | 3.3 | 0.0 | 0.0 | 0.0 | 6.1 | 17.5 | 0.0 | | 17.5 | |  |  |
| 7 | SIN | 1 | NED | 314.9 | 7.3 | 2.5 | 20.8 | 4.0 | 272.5 | 594.6 | 27.3 | | 621.9 | |  |  |
| 8 | SIN | 1 | NED | 19.4 | 2.8 | 1.1 | 0.3 | 0.0 | 83.3 | 105.6 | 1.4 | | 107.0 | |  |  |
| 10 | SIN | 2 | M→PD | 9.4 | 0.0 | 0.0 | 2.1 | 0.0 | 57.3 | 66.7 | 2.1 | | 68.7 | |  |  |
| 11 | SIN | 2 | NED | 455.5 | 21.3 | 17.0 | 145.2 | 30.2 | 175.5 | 652.4 | 192.4 | | 844.8 | |  |  |
| 12 | SIN | 2 | NED | 4.7 | 0.6 | 0.0 | 3.2 | 0.2 | 40.4 | 45.7 | 3.4 | | 49.1 | |  |  |
| 13 | SIN | 2 | NED | 3.6 | 1.6 | 1.7 | 0.6 | 0.0 | 28.4 | 33.6 | 2.3 | | 35.9 | |  |  |
| 15 | SIN | 2 | NED | 18.0 | 4.8 | 2.4 | 4.1 | 3.1 | 102.8 | 125.5 | 9.7 | | 135.2 | |  |  |
| 16 | SIN | 3 | M→PD | 16.8 | 3.3 | 0.0 | 0.7 | 0.0 | 30.3 | 50.4 | 0.7 | | 51.1 | |  |  |
| 17 | SIN | 3 | M→PD | 12.6 | 3.8 | 1.7 | 4.7 | 5.0 | 127.3 | 143.8 | 11.4 | | 155.2 | |  |  |
| 18 | SIN | 3 | M→PD | 7.2 | 4.2 | 1.8 | 1.7 | 3.0 | 38.8 | 50.2 | 6.4 | | 56.6 | |  |  |
| 19 | SIN | 3 | M→PR | 62.1 | 4.2 | 4.8 | 13.4 | 3.4 | 42.2 | 108.5 | 21.5 | | 130.0 | |  |  |
| 20 | SIN | 3 | M→SD | 53.8 | 1.1 | 1.2 | 1.2 | 0.3 | 21.2 | 76.1 | 2.7 | | 78.8 | |  |  |
| 21 | SIN | 3 | NED | 4.1 | 0.0 | 0.0 | 0.0 | 0.0 | 15.8 | 19.9 | 0.0 | | 19.9 | |  |  |
| 22 | SIN | 3 | NED | 23.8 | 5.8 | 6.2 | 58.2 | 3.9 | 73.1 | 102.6 | 68.4 | | 171.0 | |  |  |
| 23 | SIN | 3 | NED | 6.7 | 0.0 | 0.9 | 2.5 | 0.0 | 59.0 | 65.7 | 3.3 | | 69.0 | |  |  |
